# Reconstruction of Electrical Burn Sequelae Using a Muscle-Sparing Latissimus Dorsi Flap: A Case Report

**DOI:** 10.7759/cureus.88582

**Published:** 2025-07-23

**Authors:** Alhan Fernando Castillo Valencia, David Salvador Rodriguez Arevalo, Jose Maria Zepeda Torres

**Affiliations:** 1 Surgery, Universidad de Colima, Colima, MEX; 2 Plastic and Reconstructive Surgery, Unidad Medica De Alta Especialidad (UMAE) Hospital de Especialidades No. 71 Instituto Mexicano del Seguro Social (IMSS), Torreón, MEX; 3 Surgery, University of Guadalajara, Guadalajara, MEX

**Keywords:** electrical burn, latissimus dorsi flap, muscle sparing, plastic and reconstructive surgery, sequela

## Abstract

The latissimus dorsi (LD) muscle, located in the posterior thoracolumbar region, plays a crucial role in shoulder movement, including extension, adduction, and internal rotation of the humerus. Its reliable vascular anatomy and versatility make it a frequent option in reconstructive surgery for complex defects, particularly when local tissues are compromised due to severe trauma. High-voltage electrical burns often cause deep, systemic damage, requiring surgical strategies that maximize tissue coverage while minimizing functional loss. We present the case of a 54-year-old man who presented to the emergency department after sustaining a high-voltage electrical burn. The clinical course was complicated by ischemia and purulent discharge in the upper limbs and thoracic wound, despite antibiotic therapy and primary closure attempts.

A thoracic defect required debridement and was reconstructed using a muscle-sparing LD flap. This approach aimed to preserve the majority of the LD muscle and allow for faster rehabilitation for the patient.

## Introduction

Burn defects are injuries that must be managed with special care due to the pro-inflammatory state experienced by the patient and the loss of the protective barrier that is the skin. This makes the affected site prone to infection. Additionally, electrical burns cause cellular damage that is not immediately visible but becomes apparent as the affected segment evolves, revealing its sequelae. For this reason, the management of these wounds presents a challenge for reconstructive surgeons. 

The latissimus dorsi (LD) muscle, located in the posterior thoracolumbar region, plays fundamental roles in shoulder movement, such as extension, adduction, and internal rotation of the humerus. Its vascularization and versatile anatomy make it a common alternative in reconstructive surgery to treat complex defects, especially when adjacent tissues are compromised or there is a history of severe trauma [1]. In patients with high-voltage electrical burns, which often cause deep and systemic damage, surgical management requires strategies that optimize tissue coverage without compromising the patient’s residual function [2].

The muscle-sparing LD flap has emerged as an effective option that enables reconstruction with reduced donor-site morbidity, which is particularly useful in scenarios where prolonged rehabilitation is anticipated [3]. In this paper, we present the case of a patient with thoracic sequelae secondary to an electrical burn.

The muscle-sparing LD flap as an early solution was the most convenient option for the patient, given the complexity of the defect location and the history of dehiscence following primary closure. The outcome was satisfactory and significant for our center, as the coverage issue was resolved promptly using a method that can also be performed in other centers without microsurgery infrastructure. 

## Case presentation

A 54-year-old man presented to the emergency department after sustaining a high-voltage electrical burn caused by contact with a power line. He denied tobacco, alcohol, or drug use, as well as any relevant allergies.

The current injury occurred while installing a security camera near high-voltage wiring. Upon accidental contact, he experienced a high-voltage electrical discharge, resulting in third-degree burns to the palms (entry site), with upper limb involvement and an exit wound through the right posterior hemithorax, where deep second-degree burns were sustained.

**Figure 1:**
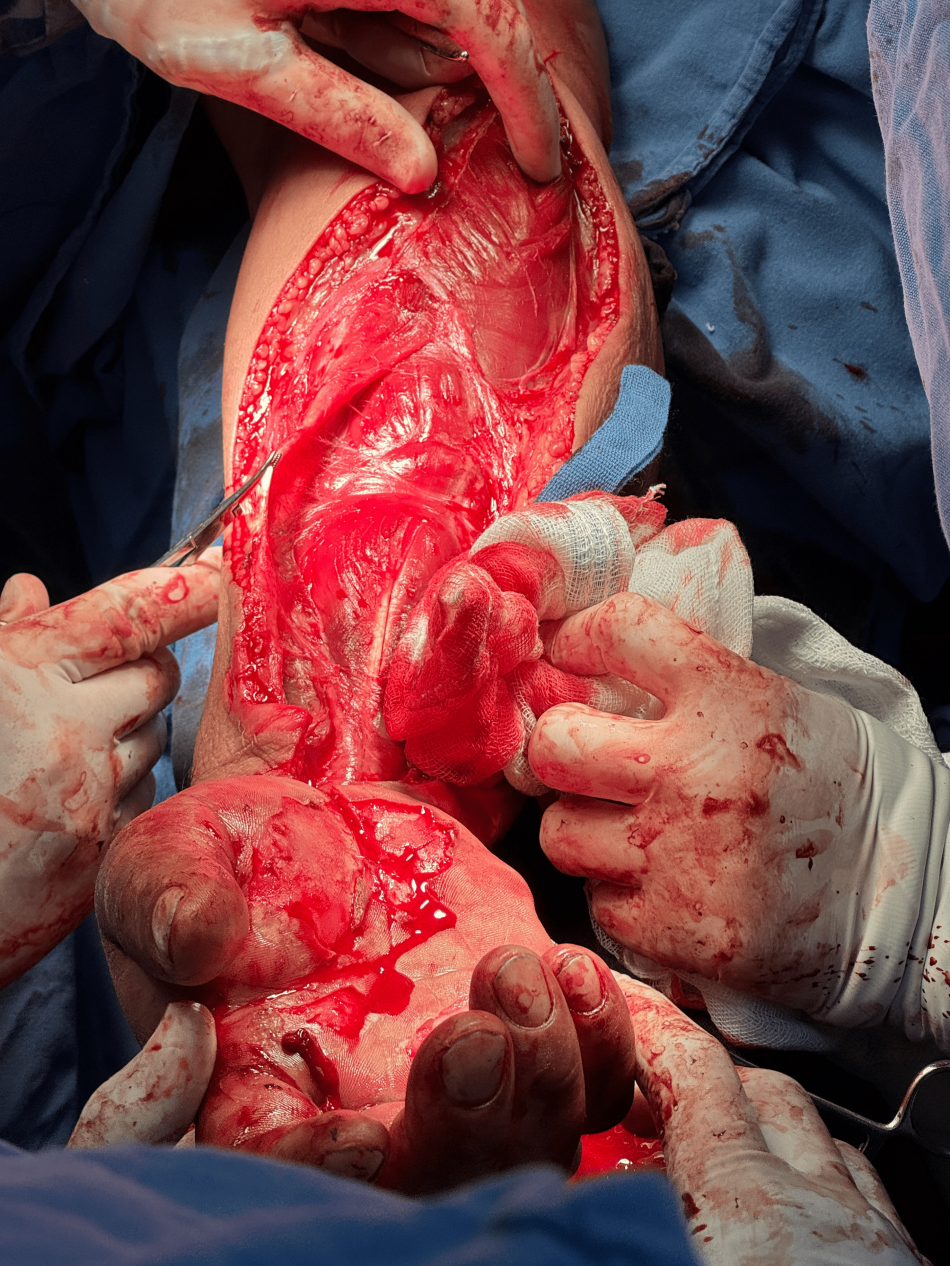
Italic S-shaped volar incision on the right upper extremity.

Initial management included fluid resuscitation and analgesia, followed by laboratory and imaging studies. The patient underwent surgical debridement and fasciotomies of both hands (Figure 1).

He was admitted to the intensive care unit, where surgical debridements continued every 48 hours for one week. Fasciotomy closure was attempted; however, clinical improvement was not observed, and evolution was unfavorable. Purulent exudate was noted from wounds on the hands and forearms, accompanied by absence of capillary refill, paresthesia, and pain on mobilization. The right thoracic burn showed signs of ischemia.

**Figure 2:**
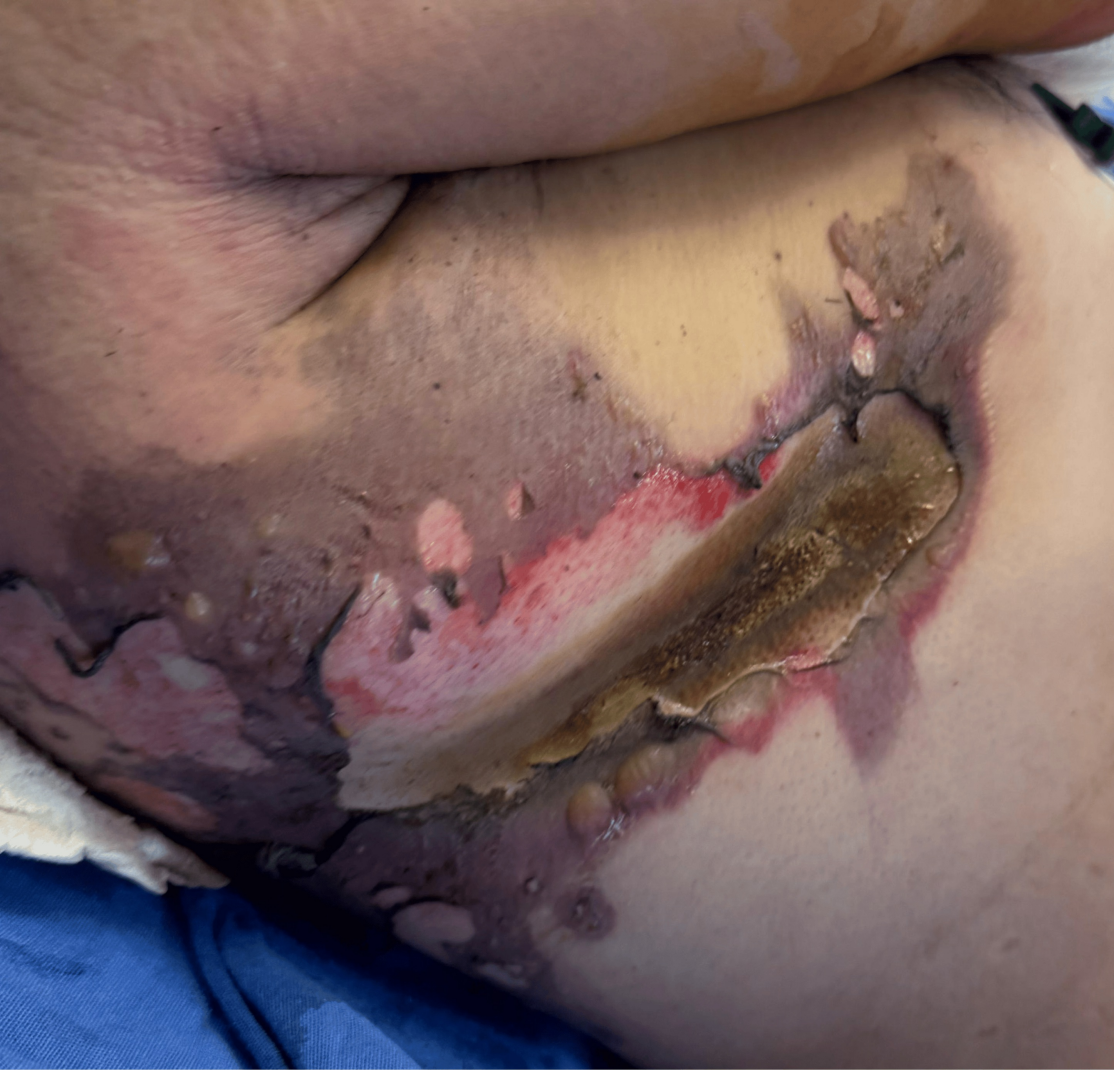
Electrical burn injury in the right posterior thoracic region

**Figure 3:**
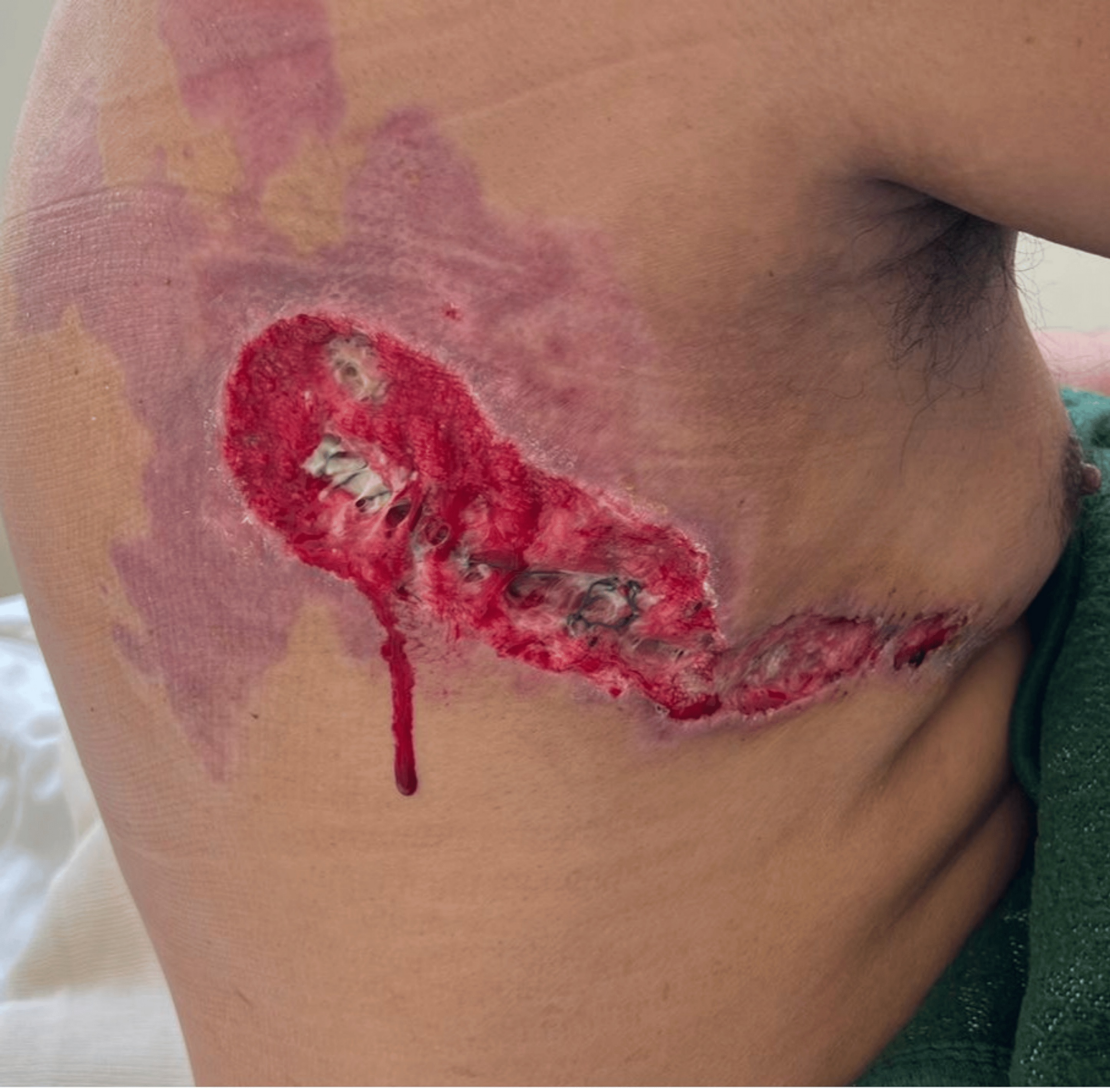
Post-debridement and primary closure wound.

Empiric antibiotic therapy with meropenem was initiated, along with wound care to the upper limbs and thoracic area. A 20 cm thoracic burn wound was debrided and closed primarily, but healing was unfavorable due to dehiscence (Figures 2, 3).

**Figure 4:**
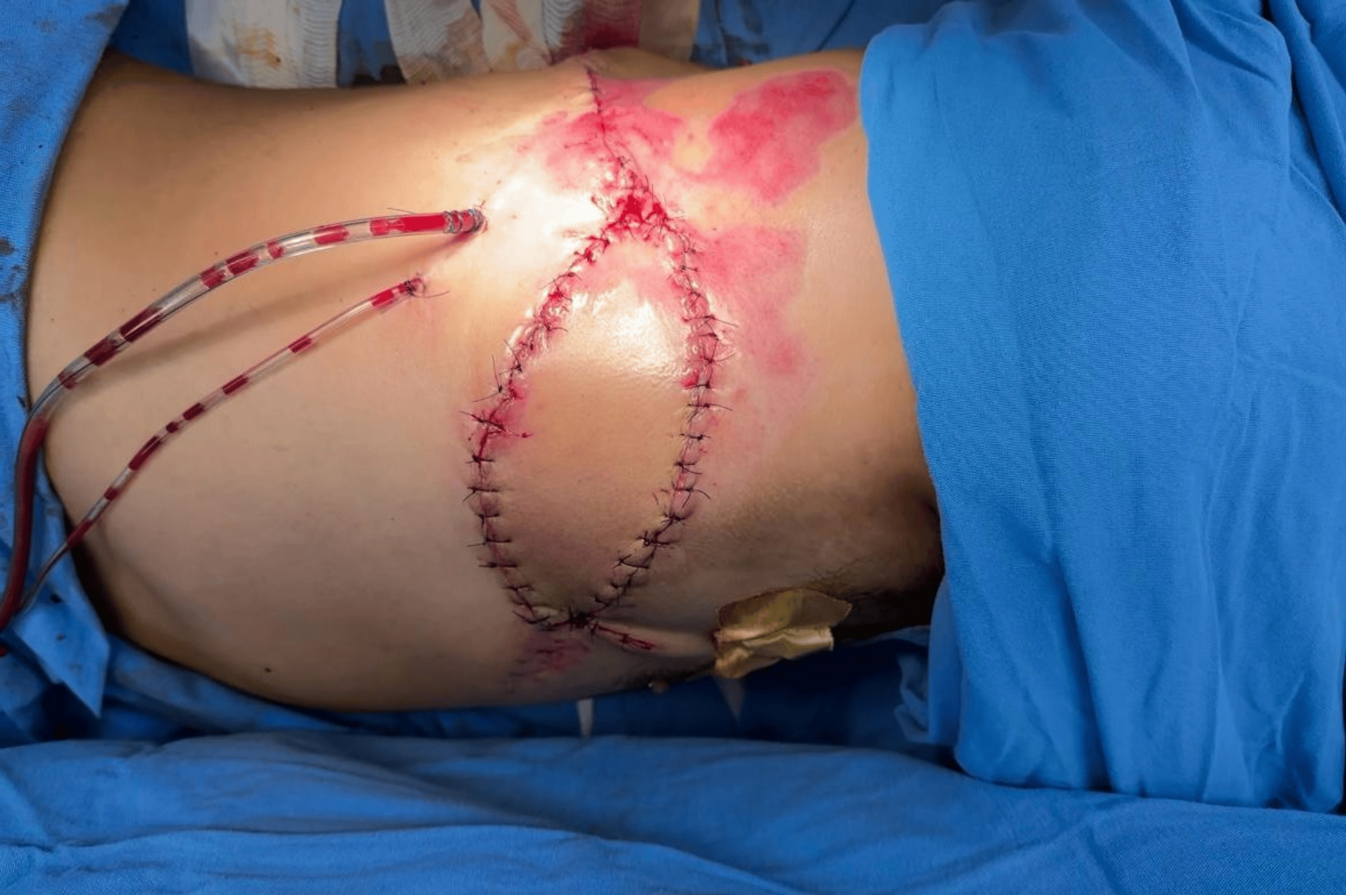
Immediate postoperative result

**Figure 5:**
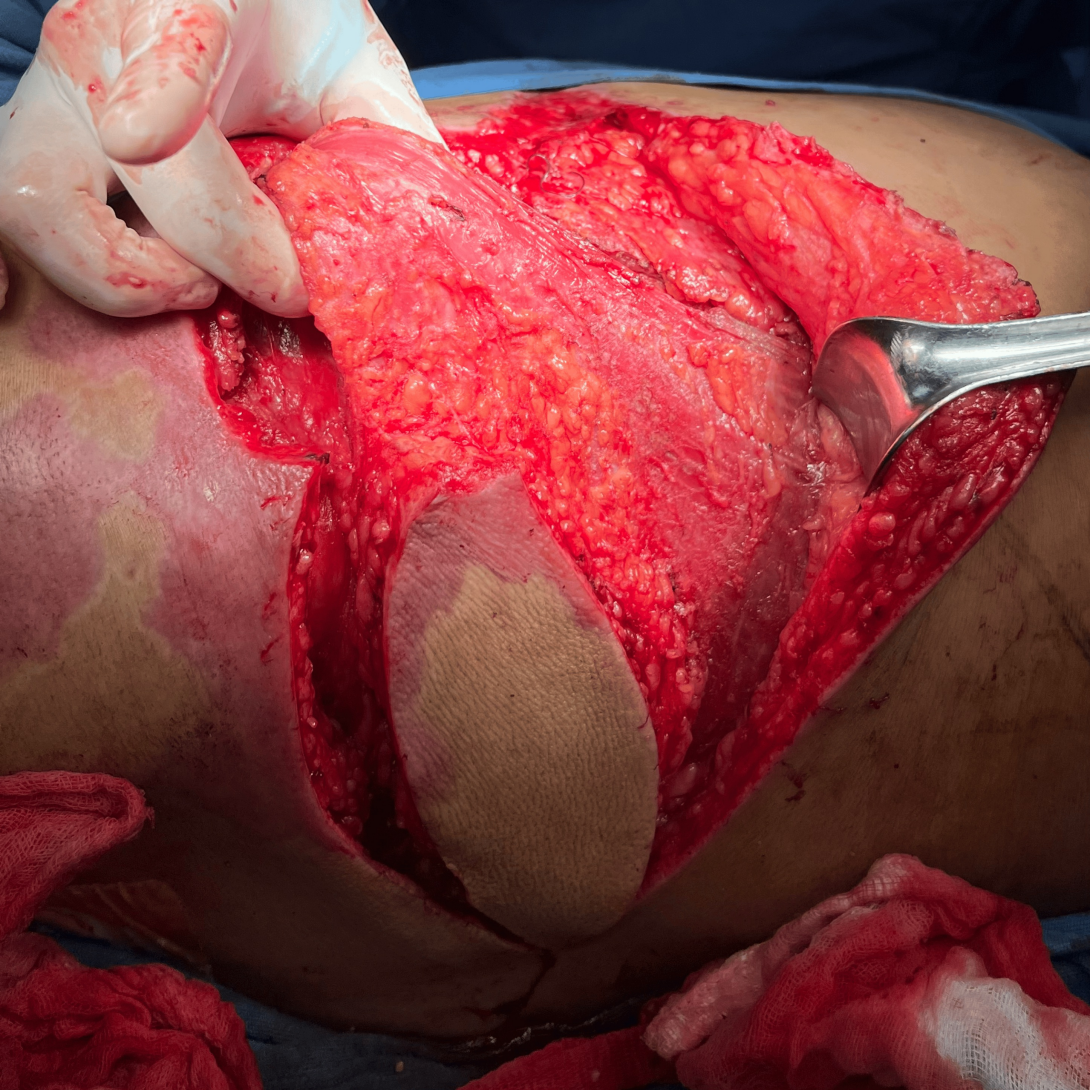
Muscle-sparing latissimus dorsi flap

The reconstructive elevator concept was applied, leading to the decision to use a flap for defect coverage. Among the available options, fasciocutaneous flaps were excluded due to their vulnerability to necrosis, limited capacity to fill deep spaces, and therefore reduced resistance to infection. Local or regional flaps were ruled out because of potential subclinical tissue compromise. Free flaps were not feasible given the need for microsurgical infrastructure, which was unavailable at our center. The LD flap was chosen, as it provides a well-vascularized muscle with a long, reliable pedicle, substantial volume, and greater resistance in necrotic environments (Figures 4, 5).

Postoperative recovery was uneventful, and the flap healed satisfactorily with no vascular compromise. The patient is currently undergoing rehabilitation and remains under outpatient follow-up by the plastic surgery team.

## Discussion

Managing high-voltage electrical burns remains a multidisciplinary challenge due to the extent and depth of tissue damage, affecting skin, subcutaneous tissue, muscle, blood vessels, and sometimes bone. Ischemic progression and frequent secondary infection necessitate continuous surgical evaluation, serial debridements, and timely decisions regarding definitive reconstruction [4].

Microsurgery may be an option when management with an LD flap is not possible. It can be performed in the acute phase or during the treatment of sequelae; however, this approach is carried out in specialized centers and is not available in most hospitals [5].

In this context, reconstruction of the affected posterior thoracic wall presents a particular challenge, as it requires durable, well-vascularized coverage with minimal functional impact in a potentially debilitated patient [6]. The muscle-sparing LD flap offers an ideal solution in such cases. Its consistent vascular supply via the thoracodorsal artery, broad arc of rotation, and substantial skin and subcutaneous tissue volume enable safe coverage of extensive defects without compromising back muscle function, which is crucial in patients requiring prolonged functional rehabilitation [7].

Compared to other reconstructive options, such as the transverse rectus abdominis muscle (TRAM) flap or pectoralis major flap, the LD flap provides greater flexibility in design, a larger skin surface, and lower donor-site morbidity. Furthermore, by avoiding microvascular anastomoses, this technique reduces operative time and potential vascular complications in patients already burdened by systemic stress from electrical trauma [8].

The concept known as the “reconstructive ladder”, originally introduced by Sir Harold Gillies, was later refined and widely disseminated by Mathes and Nahai. This model offers a systematic approach for selecting surgical techniques, progressing from less to more complex interventions. It prioritizes reconstructive strategies based on the level of technical difficulty required, with the goal of ensuring the safest and most appropriate outcome for the patient [9].

This case underscores the value of the LD flap within the "reconstructive elevator" concept, which advocates for rational, early selection of the most appropriate technique based on defect location, severity, and patient condition. This approach prioritizes not only anatomical restoration, but also functional, aesthetic, and psychological recovery [9].

## Conclusions

The muscle-sparing LD flap is an effective and versatile reconstructive option for managing posterior thoracic defects caused by high-voltage electrical burns. It is important for the reconstructive surgeon to consider the concept of the reconstructive elevator, which emphasizes the need for advanced techniques to address complex defects. The ultimate goal is always to tailor the best strategy by individualizing care for each patient. 

Unlike other flaps, a muscle-sparing LD flap provides reliable coverage for deep defects, and its robust vascularity makes it particularly suitable for areas with adjacent ischemic damage. Its use promotes early functional recovery, minimizes donor-site morbidity, and ensures dependable anatomical coverage. Microsurgical flap options were not feasible due to limitations in our hospital’s infrastructure; however, they may be considered in future similar cases. This case supports the rational use of this technique as part of a personalized reconstructive strategy that enhances overall patient outcomes and facilitates early rehabilitation. 
